# 2194: Effects of anoxia on viability and differentiation of human cardiosphere-derived cells

**DOI:** 10.1017/cts.2017.28

**Published:** 2018-05-10

**Authors:** Michael Khanjyan, Vien Nguyen, Eric Kazangian, Shane Browne, Kevin Healy, Kurosh Ameri, Yerem Yeghiazarians

## Abstract

OBJECTIVES/SPECIFIC AIMS: A major limitation of cardiac stem cell transplantation following myocardial infarction (MI) is poor retention of cells in the ischemic microenvironment. Our study aims to better understand and promote the survival and differentiation of human cardiosphere-derived cells (hCDCs) in anoxia, a feature of infarcted myocardium. METHODS/STUDY POPULATION: We previously demonstrated that TGFβ1 and heparin-containing hydrogels (TH-hydrogel) can promote murine CDC survival. In this study, hCDCs were incubated in either normoxia or anoxia for 8 hours with and without TH-hydrogel. In addition, hCDCs without TH-hydrogel were assessed in 16 hours of anoxia. Following incubation, hCDCs were assayed for viability using calcein dye and immunostained for CD31, a marker of endothelial differentiation. RESULTS/ANTICIPATED RESULTS: hCDCs incubated for 8 hours in anoxia in both models equally demonstrated increased survival up to 30% when compared with cells incubated in normoxia. However, in contrast to hCDCs alone, hCDCs with TH-hydrogel additionally demonstrated increased differentiation into endothelial cells in both anoxia and normoxia. We found that hCDCs alone were able to upregulate CD31 only when subjected to 16 hours of anoxia. DISCUSSION/SIGNIFICANCE OF IMPACT: We demonstrate a new, previously unknown response of hCDCs to anoxia. This induces increased viability and differentiation of hCDCs into endothelial cells. The differentiation in anoxia was time dependent and could be expedited with use of TH-hydrogel. Anoxic preconditioning of hCDCs together with the TH-hydrogel system may improve the therapeutic potential of stem cell transplantation following MI.

